# Sex-specific differences in cardiac transthyretin amyloidosis: addressing the diagnostic gap in women

**DOI:** 10.1093/ehjopen/oeaf175

**Published:** 2025-12-26

**Authors:** Julia Vogel, Sophia Jura, Stephan Settelmeier, Tobias Lerchner, Florian Buehning, Loubna Yahsaly, Alexander Carpinteiro, H Christian Reinhardt, Tienush Rassaf, Lars Michel

**Affiliations:** Department of Cardiology and Vascular Medicine, West German Heart and Vascular Center, University Hospital Essen, Hufelandstr. 55, Essen 45147, Germany; West German Amyloidosis Center, University Hospital Essen, Hufelandstr. 55, Essen 45147, Germany; Department of Cardiology and Vascular Medicine, West German Heart and Vascular Center, University Hospital Essen, Hufelandstr. 55, Essen 45147, Germany; West German Amyloidosis Center, University Hospital Essen, Hufelandstr. 55, Essen 45147, Germany; Department of Cardiology and Vascular Medicine, West German Heart and Vascular Center, University Hospital Essen, Hufelandstr. 55, Essen 45147, Germany; West German Amyloidosis Center, University Hospital Essen, Hufelandstr. 55, Essen 45147, Germany; Department of Cardiology and Vascular Medicine, West German Heart and Vascular Center, University Hospital Essen, Hufelandstr. 55, Essen 45147, Germany; Department of Cardiology and Vascular Medicine, West German Heart and Vascular Center, University Hospital Essen, Hufelandstr. 55, Essen 45147, Germany; Department of Cardiology and Vascular Medicine, West German Heart and Vascular Center, University Hospital Essen, Hufelandstr. 55, Essen 45147, Germany; West German Amyloidosis Center, University Hospital Essen, Hufelandstr. 55, Essen 45147, Germany; Department of Hematology and Stem Cell Transplantation, West German Cancer Center, University Hospital Essen, Hufelandstr. 55, Essen 45147, Germany; West German Amyloidosis Center, University Hospital Essen, Hufelandstr. 55, Essen 45147, Germany; Department of Hematology and Stem Cell Transplantation, West German Cancer Center, University Hospital Essen, Hufelandstr. 55, Essen 45147, Germany; Department of Cardiology and Vascular Medicine, West German Heart and Vascular Center, University Hospital Essen, Hufelandstr. 55, Essen 45147, Germany; West German Amyloidosis Center, University Hospital Essen, Hufelandstr. 55, Essen 45147, Germany; Department of Cardiology and Vascular Medicine, West German Heart and Vascular Center, University Hospital Essen, Hufelandstr. 55, Essen 45147, Germany; West German Amyloidosis Center, University Hospital Essen, Hufelandstr. 55, Essen 45147, Germany

**Keywords:** Amyloidosis, ATTR, Cardiomyopathy, Gender, Heart failure, Transthyretin

## Abstract

**Aims:**

Transthyretin amyloid cardiomyopathy (ATTR-CM) is an underdiagnosed cause of heart failure. With a male:female ratio of approximately 10:1, current evidence including diagnostic criteria is largely based on male-dominant collectives. Sex-specific differences may contribute to delayed diagnosis in women. This study investigates these differences in a real-world setting.

**Methods and results:**

In this retrospective single-centre cohort study, all patients at West German Amyloidosis Center diagnosed with ATTR-CM between 2018 and 2024 were analysed. Clinical, echocardiographic, and laboratory parameters as well as outcomes under transthyretin stabilizer therapy at 6 and 12 months were evaluated. Among 240 patients, 34 (14.2%) were women. Compared to men, women had lower interventricular septal diameter, left ventricular mass, and stroke volume, but higher ejection fraction. Troponin I levels were lower and renal function was worse in women. Diagnostic delay was significantly longer in women (median 750 vs. 86 days, *P* = 0.022). Despite therapy, sex-specific echocardiographic differences persisted, and functional capacity remained lower in women, although NYHA functional class was comparable.

**Conclusion:**

Transthyretin amyloid cardiomyopathy presents with persistent sex-specific differences that may contribute to diagnostic delay in women. Current diagnostic thresholds may not adequately reflect female disease patterns, underscoring the need for sex-adapted diagnostic criteria to improve early detection and management.

## Introduction

Transthyretin amyloid cardiomyopathy (ATTR-CM) is a progressive infiltrative cardiomyopathy characterized by the deposition of misfolded transthyretin amyloid fibrils in the myocardium.^1^ Transthyretin amyloid cardiomyopathy can result from either a wild-type (ATTRwt) or variant (ATTRv) TTR gene with distinct disease phenotypes.^2^ Diagnosis of ATTR-CM relies on suggestive symptoms, so-called *red flag* symptoms and imaging modalities, with interventricular septum diameter (IVSD) ≥ 12 mm serving as a key criterion.^3^ Currently, transthyretin stabilizers represent the only approved disease-modifying therapy for ATTR-CM^4,5^ while novel therapeutic approaches including gene silencers and antibodies are under investigation.^6^

Unfortunately, the diagnosis of ATTR-CM is often delayed due to its overlapping symptoms with common heart failure. While studies have shown that the time to ATTR-CM diagnosis has improved in recent years, nearly 1 year still passes from the onset of symptoms until patients receive therapy.^7^ Approximately 13% of HFpEF patients have underlying cardiac amyloidosis,^8^ yet the condition remains underrecognized. While women are more frequently diagnosed with HFpEF, they appear to be underrepresented in ATTR-CM diagnoses.^9^ Most large studies to date have predominantly included male patients, which may have contributed to diagnostic criteria and disease recognition patterns that are more reflective of the male phenotype.^5,10^ Sex-specific differences in cardiac anatomy and normative values may further obscure diagnosis, making early detection particularly challenging in female patients. Although individual studies have shown that at the time of diagnosis, women are older, have a higher New York Heart Association (NYHA) functional class, and more frequently present with red flag symptoms like carpal tunnel syndrome and aortic valve stenosis, there is currently no standardized recommendation to individualize or alter diagnostic criteria in screening of female patients.^11^ Recently, no overt sex-specific differences were observed in a large, well-characterized ATTR-CM cohort, but a significant risk of selection bias by delayed or missed diagnosis in women remains.^12^

This study examines sex-specific differences in ATTR-CM and their impact on diagnostic timelines, echocardiographic findings, and clinical outcomes over time, aiming to provide the basis for establishing sex-specific diagnostic criteria to enhance early detection in women.

## Material and methods

We conducted a retrospective cohort study including all patients who presented with suspected ATTR-Amyloidosis at the West German Amyloidosis Center between 2018 and 2024. Inclusion criteria contained confirmed ATTR-CM via scintigraphy or endomyocardial biopsy. To exclude AL amyloidosis, all patients underwent serum and urine free light chain quantification and immunofixation. Only cases without monoclonal gammopathy or abnormal κ/λ ratio were included. Baseline demographics, echocardiographic parameters, laboratory values, and 6- and 12-month outcomes were collected. Echocardiographic assessments were conducted in our echo core laboratory, with IVSD measured by standardized protocols within our centre, aligned with the recommendations of the European Society of Cardiology (ESC) guidelines.^13^ All echocardiographic measurements were performed by experienced echocardiographers using a single echocardiography machine. Measurements followed standardized acquisition protocols and were reviewed independently. Disease stage was classified according to the National Amyloidosis Centre (NAC) staging system^14^ which stratifies patients based on NT-proBNP and eGFR: Stage I: NT-proBNP ≤ 3000 pg/mL and eGFR ≥ 45 mL/min/1.73 m², Stage II: NT-proBNP > 3000 pg/mL or eGFR < 45 mL/min/1.73 m², Stage III: NT-proBNP > 3000 pg/mL and eGFR < 45 mL/min/1.73 m², and Stage IV: NT-proBNP > 10 000 pg/mL. The primary endpoints of this study were defined to evaluate sex-specific differences in the diagnosis, clinical presentation, and treatment response in patients with ATTR-CM. The primary endpoint of this study was the diagnostic delay, defined as the time from first documented symptom or identifiable abnormal finding (e.g. echocardiography, MRI, or scintigraphy) to the confirmed diagnosis of ATTR-CM. Diagnostic delay was assessed exclusively on the basis of individual patient-level time intervals. Data were systematically gathered from the clinic’s internal data system. Statistical analyses included two-sided *t*-tests and Mann–Whitney *U* tests for continuous variables and χ² tests for dichotomous variables. Normality was assessed using the Shapiro–Wilk test. A *P* < 0.05 was considered statistically significant. Ethics approval was obtained from the University of Duisburg-Essen (23-11500-BO). This study is reported in accordance with the STROBE (Strengthening the Reporting of Observational Studies in Epidemiology) guidelines (Supplementary material online, *Table S1*).

## Results

### Baseline characteristics

**Figure 1:**
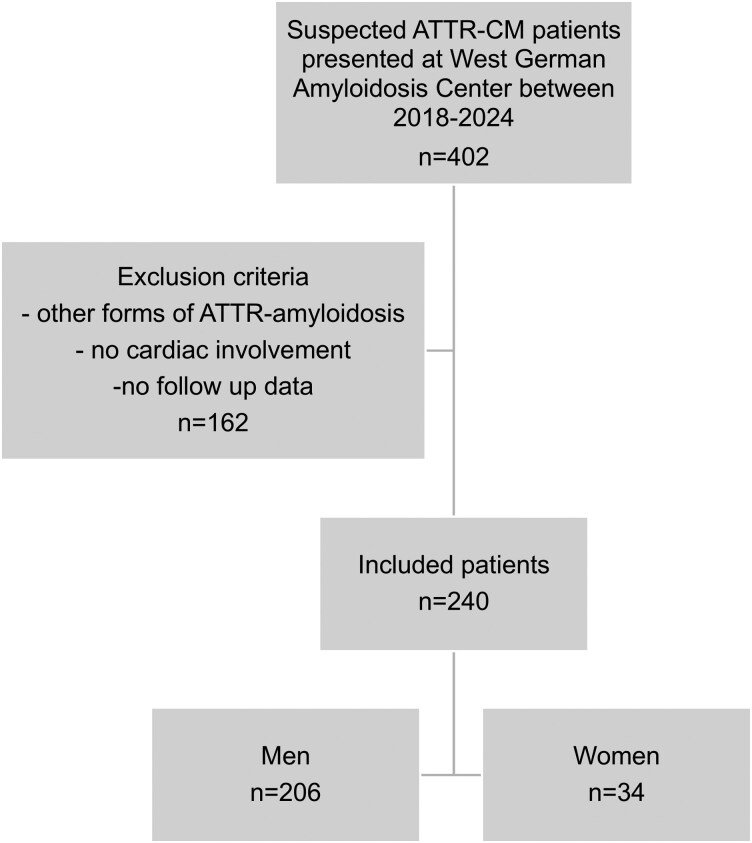
Flow diagram of patient selection. ATTR, transthyretin amyloidosis; CM, cardiomyopathy.

Of the 402 screened patients, 162 were excluded: 78 due to incomplete evaluation or absence of follow-up at our centre and 84 because no cardiac involvement was present at the time of assessment. These cases therefore did not meet diagnostic criteria for ATTR-CM and lacked clinical or echocardiographic data comparable to the included cohort (*Figure 1*). The median age was 80 years (76-83): in men 80 years (76-83) and in women 82 years (78-84). Overall, 34 patients (14.2%) were women and 206 (85.8%) were men. Most patients were diagnosed with ATTRwt (96.2%). Mean 6 min walk distance (6MWD) was 286 ± 97 m and most patients were in NYHA functional class I + II [132 (57.9%)]. There were sex-specific differences in baseline characteristics: coronary artery disease was significantly more prevalent among men (32.4% vs. 53.4%, *P* = 0.023), whereas women had a higher prevalence of carpal tunnel syndrome in their medical history (58.8% vs. 40.8%, *P* = 0.049) (*Table 1*).

**Table 1:** Baseline characteristics

Variable	All	Women	Men	*P*-value
	*n* = 240	*n* = 34	*n* = 206	
Age (years)	80 (76–83)	82 (78–84)	80 (76–83)	0.071
BSA (m^2^)	2.0 (1.9–2.1)	1.7 (1.6–1.8)	2.0 (1.9–2.1)	<0.001^a^
NYHA functional class, *n* (%)				0.855
1 + 2	132 (57.9)	19 (59.4)	113 (57.7)	
3 + 4	96 (42.1)	13 (40.6)	83 (42.3)	
6 min walk distance (m)	286 ± 97	256 ± 72	292 ± 101	0.169
Coronary artery disease, *n* (%)	121 (50.4)	11 (32.4)	110 (53.4)	0.023^a^
Atrial fibrillation, *n* (%)	158 (65.8)	21 (61.8)	137 (66.5)	0.589
Carpal tunnel syndrome, *n* (%)	104 (43.3)	20 (58.8)	84 (40.8)	0.049^a^
Polyneuropathy, *n* (%)	58 (24.4)	11 (32.4)	47 (23.0)	0.242
Aortic valve stenosis, *n* (%)	8 (3.4)	0 (0)	8 (3.9)	0.239
Medication, *n* (%)				
Beta-blocker	141 (58.8)	23 (67.7)	118 (57.3)	0.255
ACE inhibitor/AT1-antagonist	134 (55.8)	24 (70.6)	110 (53.4)	0.062
ARNi	12 (5.0)	0 (0)	12 (5.8)	0.149
MR-antagonist	99 (41.2)	12 (35.3)	87 (42.2)	0.446
SGLT-2-inhibitor	62 (25.8)	12 (35.3)	50 (24.3)	0.174
Diuretics	172 (71.7)	23 (67.7)	149 (72.3)	0.575
OAC	158 (65.8)	20 (58.8)	138 (67.0)	0.352
Transthyretin stabilizer	226 (94.2)	30 (88.2)	196 (95.2)	0.111
Amyloidosis types, *n* (%)				0.008^a^
ATTRwt	231 (96.2)	30 (88.2)	201 (97.6)	
ATTRv	9 (3.8)	4 (11.8)	5 (2.4)	
NAC staging, *n* (%)				0.452
* <2*	98 (41.2)	12 (35.3)	86 (42.2)	
≥*2*	140 (58.8)	22 (64.7)	118 (57.8)	

ACE, angiotensin-converting enzyme; ARNi, angiotensin-receptor-neprilysin-inhibitor; AT1, angiotensin type 1; ATTRv, variant transthyretin amyloidosis; ATTRwt, wild-type transthyretin amyloidosis; BSA, body surface area; MRA, mineralocorticoid receptor antagonists; NAC, national amyloidosis centre; NYHA, New York Heart Association; OAC, oral anticoagulation; SGLT-2, sodium-glucose co-transporter 2.

^a^ *P* < 0.05.

Laboratory parameters also revealed sex-specific variations. Women had significantly lower high-sensitive troponin I (hs-cTnI) levels at the time of diagnosis compared to men [30 (14–81) ng/L vs. 45 (27–72) ng/L, *P* = 0.042], but exhibited poorer renal function, with a lower estimated glomerular filtration rate (eGFR) (53.2 ± 18.8 mL/min/1.73m² vs. 60.1 ± 18.6 mL/min/1.73m², *P* = 0.046). Prevalence of atrial fibrillation was similar between sexes (*P* = 0.589) (*Table 2*).

**Table 2:** Baseline laboratory values and echocardiography parameters

Variable	All	Women	Men	*P*-value
	*n* = 240	*n* = 34	*n* = 206	
Laboratory values				
hs-cTnI (ng/L)	46 (26–73)	30 (14–81)	45 (27–72)	0.042^a^
NT-proBNP (pg/mL)	3171 (1660–5663)	3046 (907–4800)	3216 (1686–6065)	0.136
Creatinine (mg/dL)	1.1 (1.0–1.4)	1.1 (0.8–1.3)	1.2 (1.0–1.4)	0.057
eGFR (mL/min/1.73m^2^)	59.1 ± 18.8	53.2 ± 18.8	60.1 ± 18.6	0.046^a^
CRP (mg/dL)	0.4 (0.4–0.5)	0.4 (0.4–0.9)	0.4 (0.4–0.5)	0.287
Hb (g/dL)	13.6 (12.4–14.6)	13.2 (12.2–14.1)	13.6 (12.5–14.9)	0.039^a^
Echocardiography				
LVEF (%)	52 (45–55)	55 (50–60)	52 (43–55)	<0.001^a^
LVEDD (mm)	45 (41–50)	41 (38–46)	45 (41–50)	0.016^a^
LVMMi (g/m^2^)	305 (249–374)	124 (94–190)	161 (132–189)	0.104
LAVI (mL/m^2^)	51 (36–61)	42 (36–56)	52 (36–61)	0.406
TAPSE (mm)	17.0 (13.7–20.8)	17.1 (15.5–20.3)	17 (13.5–20.8)	0.670

CRP, C-reactive peptide; eGFR, estimated glomerular filtration rate; Hb, haemoglobin; hs-cTnI, high-sensitive cardiac troponin I; INR, international normalized ratio; IVSD, interventricular septum diameter; LAVI, left atrial volume index; LVEDD, left ventricular end-diastolic diameter; LVEF, left ventricular ejection fraction; LVMMi, left ventricular myocardial mass indexed to body surface area; NT-proBNP, *n*-terminal brain natriuretic peptide; TAPSE, tricuspid annular plane systolic excursion.

^a^ *P* < 0.05.

At baseline, 94% of patients received tafamidis, which was the only TTR stabilizer used in this cohort. Treatment rates were comparable between men and women (*P* = 0.74). In total, 7.8% initiated the 20 mg formulation prior to February 2020, while all later prescriptions were 61 mg. The remaining 6% did not receive a stabilizer due to frailty, late clinical presentation, or patient preference. Overall discontinuation occurred in 16.2% of treated patients, most frequently due to frailty or clinical progression/patient decision, without sex-specific differences in any treatment parameter (all *P* > 0.05; Supplementary material online, *Table S2*).

Concomitant cardiovascular medication, including beta-blockers, ACE inhibitors or ARBs, MRAs, and diuretics, was documented at baseline and is summarized in *Table 1*. No sex-related differences in background therapy were observed.

### Echocardiographic parameters

**Figure 2:**
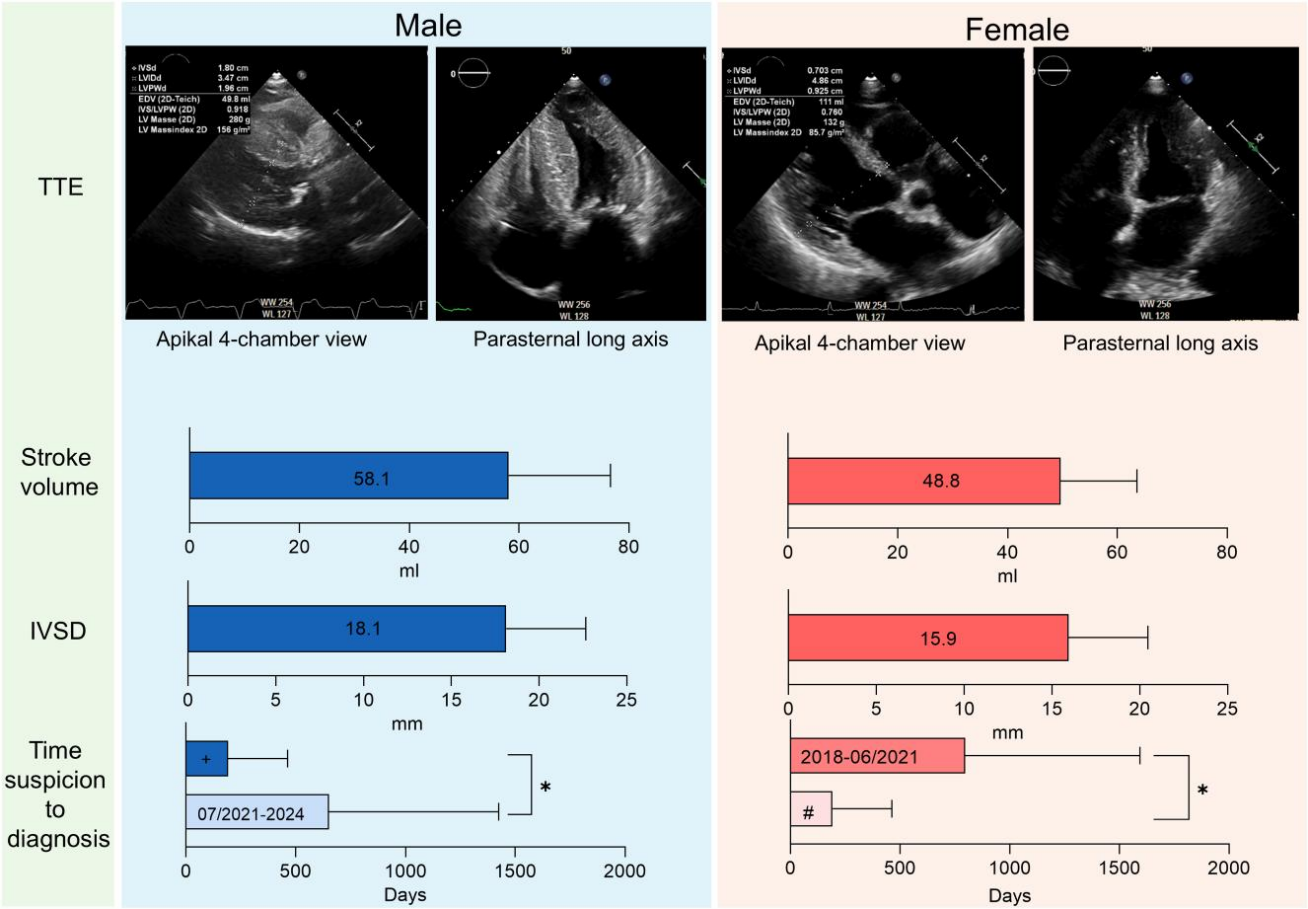
Echocardiographic and time from suspicion to diagnosis differences between women and men. IVSD, interventricular septum diameter; TTE, transthoracic echocardiography; *P < 0.05; ^+^2018–06/2021; ^#^07/2021–2024. Mean ± SD.

Echocardiographic findings showed further sex-specific differences. Women had a higher left ventricular ejection fraction (LVEF) [55 (50–60) % vs. 53 (43–55) %, *P* < 0.001] and a lower IVSD (16.2 ± 4.5 mm vs. 18.1 ± 4.5 mm, *P* = 0.026). Additionally, women exhibited a lower stroke volume (SV) compared to men [49 (42–49) mL vs. 56 (46–69) mL, *P* = 0.020]. The left ventricular myocardial mass indexed to body surface area (LVMMi) showed no differences [124 (93–190) g/m^2^ vs. 161 (132–189) g/m^2^, *P* = 0.10)] (*Figure 2* and *Table 2*).

### Time to diagnosis

We analysed the time intervals from the initial indication of disease, such as unexplained dyspnoea, abnormal findings on echocardiography, or incidental abnormalities detected through MRI or scintigraphy, to the confirmed diagnosis. In 54 patients, this analysis could not be conducted due to insufficient documentation of the first identifiable abnormality. For descriptive purposes, *Figure 2* illustrates median time from first clinical suspicion to diagnosis by sex and calendar period. All formal analyses of diagnostic delay were based on individual patient-level intervals from symptom onset or first abnormal finding to confirmed diagnosis. To avoid heterogeneity in documentation, we defined a standardized primary delay metric (symptom onset to diagnosis) and performed formal delay analyses. In the subgroup with complete symptom-onset data (*n* = 185), median diagnostic delay did not differ significantly between sexes [men: 273 days (IQR 73–744) vs. women: 312 days (IQR 121–1050), *P* = 0.421]. Sensitivity analyses using the interval from first abnormal imaging to diagnosis also showed no sex-specific differences [men: 67 days (14–182) vs. women: 38 days (19.8–172), *P* = 0.808]. A Kaplan–Meier analysis confirmed the absence of sex-specific differences in time to diagnosis (log-rank *P* = 0.387). The 54 patients with missing onset dates showed no sex imbalance and were excluded from delay analyses (see Supplementary material online, *Figure S1*).

### Multivariable linear regression

To address potential confounding as requested by the reviewer, we performed multivariable linear regression models including the following covariates: age, body surface area, ATTR subtype, NAC stage, coronary artery disease, and atrial fibrillation. In the adjusted models, the association between female sex to interventricular septal thickness (β −1.72 mm, 95% CI −4.05–0.61, *P* = 0.15) and lower LVMMi was attenuated (β −38.4 g/m², 95% CI −92.7–15.8, *P* = 0.17; Supplementary material online, *Table S3*). Effect directions were consistent with unadjusted analyses. Additional sensitivity analyses using alternative indexation approaches yielded consistent results and are shown in Supplementary material online, *Table S4*.

### Follow-up analysis

**Figure 3:**
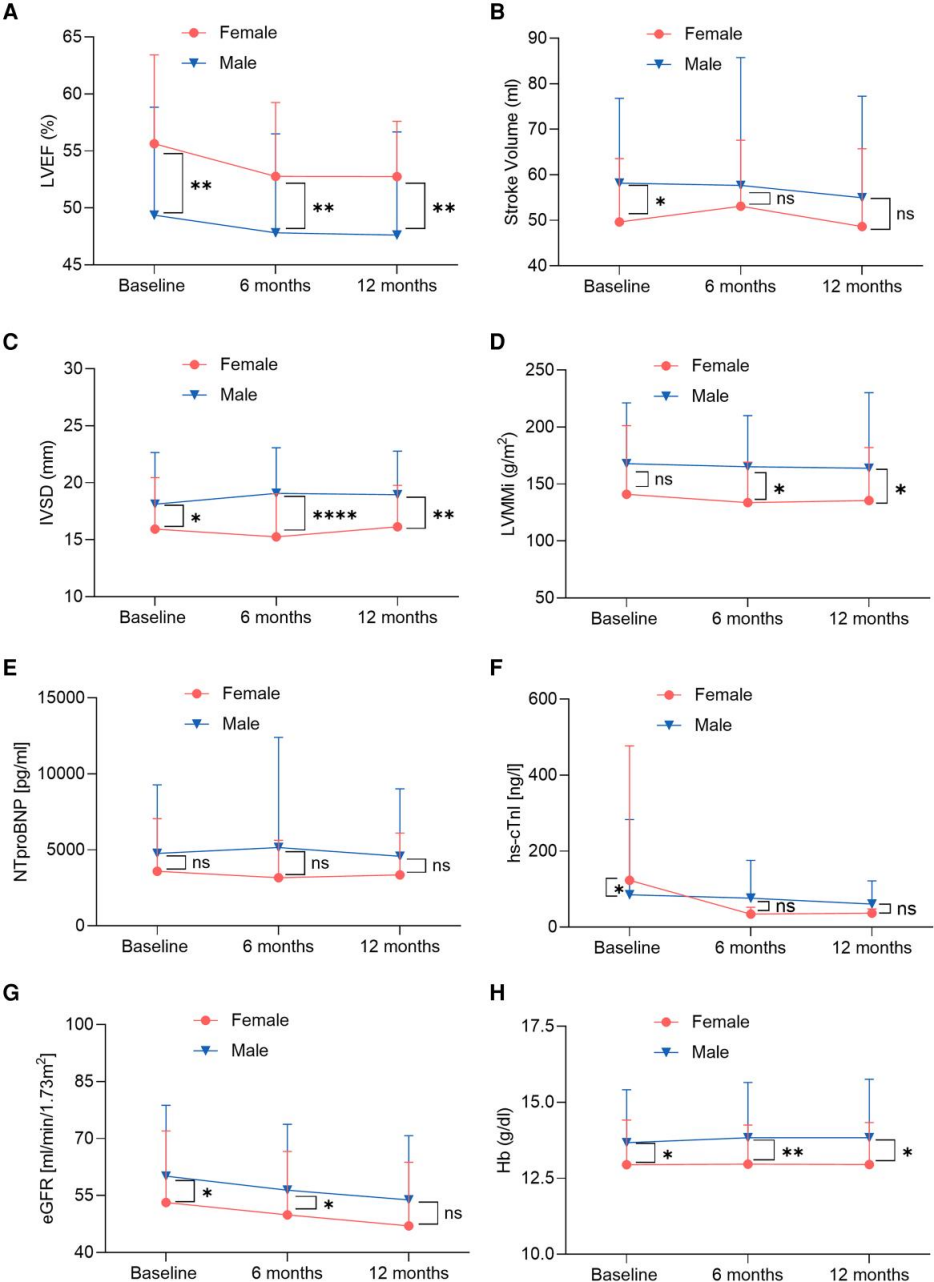
Echocardiographic and laboratory values in female and male ATTR-CM patients at baseline and follow-up. (*A*) LVEF at baseline and at FU; (*B*) SV at baseline and at FU; (*C*) IVSD at baseline and at FU; (*D*) LVMMi at baseline and at FU; (*E*) NT-proBNP at baseline and at FU; (*F*) hs-cTnI at baseline and at FU; (*G*) eGFR at baseline and at FU; (*H*) Hb at baseline and at FU. ATTR-CM, transthyretin amyloidosis cardiomyopathy; eGFR, estimated glomerular filtration rate; FU, follow-up; Hb, haemoglobin; hs-cTnI, high-sensitive cardiac troponin I; IVSD, interventricular septum diameter; LVEF, left ventricular ejection fraction; LVMMi, left ventricular myocardial mass indexed to body surface area; NT-proBNP, N-terminal brain natriuretic peptide; SV, stroke volume. For normally distributed data, two-way ANOVA was used; for non-normally distributed data, the Kruskal–Wallis test was applied. *P < 0.05, **P < 0.01, ***P < 0.001, ****P < 0.0001.

All parameters were assessed at 6 and 12 months, revealing persistent differences between women and men. Left ventricular ejection fraction remained higher in women at both 6 and 12 months, consistent with baseline findings, with a median of 55% (50–55%) compared to men, who exhibited an LVEF of 50% (42–55%) at 6 months (*P* = 0.001) and 50% (40–55%) at 12 months (*P* = 0.003). This pattern indicates that women with diagnosed ATTR-CM maintain a slightly higher LVEF over time under stabilizer therapy. Interventricular septum diameter differences persisted, with women presenting a mean IVSD of 15.2 ± 3.8 mm at 6 months compared to 19.1 ± 4.0 mm in men (*P* < 0.001). This disparity remained evident at 12 months, with women showing an IVSD of 16.1 ± 3.6 mm vs. 19.0 ± 3.8 mm in men (*P* = 0.003) (*Figure 3*).

Differences were also observed in the LMMi over time. At baseline, no difference was noted between women and men, with median LVMMi values of 124 g/m² (94–190 g/m²) and 161 g/m² (132–189 g/m²), respectively (*P* = 0.104). However, differences emerged during follow-up: at 6 months, women had a median LVMMi of 141 g/m² (98–158 g/m²) compared to 163 g/m² (136–186 g/m²) in men (*P* = 0.017). This divergence persisted at 12 months, with women exhibiting a median LVMMi of 130 g/m² (129–150 g/m²), while men showed higher values of 168 g/m² (139–207 g/m²) (*P* = 0.013) (*Figure 3*). In contrast, no significant sex differences were observed over time in stroke volume (6 months: *P* = 0.897, 12 months: *P* = 0.388) or in left atrial size, assessed by left atrial volume index (LAVI; 6 months: *P* = 0.545, 12 months: *P* = 0.057).

Renal function was already poorer in women than in men at baseline and remained different after 6 months, with women exhibiting a mean eGFR of 49.9 ± 16.7 mL/min/1.73m² compared to 56.4 ± 17.3 mL/min/1.73m² in men (*P* = 0.047). Regarding cardiac biomarkers, no significant differences were observed over the follow-up period for troponin (6 months: *P* = 0.361, 12 months: *P* = 0.595) or NT-proBNP (6 months: *P* = 0.246, 12 months: *P* = 0.204) (*Figure 3*). Clinical exercise capacity remained lower in women after 1 year, as demonstrated by a shorter 6 min walk distance (6MWT) compared to men [women: 256 m (178–276 m) vs. men: 321 m (267–368 m), *P* = 0.006). However, there were no significant differences between women and men in NYHA functional class at either 6 months (*P* = 0.822) or 12 months (*P* = 0.915).

When restricting analyses to the ATTRwt subgroup (*n* = 218), women continued to show lower interventricular septal thickness (16.1 ± 3.9 mm vs. 18.4 ± 4.2 mm, *P* = 0.018) and lower LV mass index (128 ± 41 g/m² vs. 162 ± 49 g/m², *P* = 0.021) compared to men, with LVEF remaining higher (55 ± 6% vs. 51 ± 8%, *P* = 0.032) (see Supplementary material online, *Figure S2*).

## Discussion

This study demonstrates pronounced sex-specific differences in the diagnosis, disease progression, and echocardiographic characteristics of ATTR-CM. Women were diagnosed significantly later than men, despite the presence of established red flags. Echocardiographic findings consistently showed lower IVSD, LVMMi, and SV in women, while LVEF remained higher. These disparities persisted under transthyretin stabilizer therapy at 6 and 12 months.

The observed diagnostic delay in women underscores a critical gap in early recognition of ATTR-CM. Our findings align with previous studies indicating that older women with HFpEF may be underdiagnosed for ATTR-CM. This delay may be attributed to sex-specific cardiac structural differences, lower baseline troponin levels, and the potential misattribution of symptoms to other cardiovascular conditions. Despite improvements in diagnostic rates over recent years, the median time from symptom onset to diagnosis remains markedly longer for women, reinforcing the need for enhanced diagnostic strategies tailored to female patients.

Beyond diagnostic challenges, the previously included statement suggesting that these persistent echocardiographic differences could imply distinct therapeutic considerations has been removed. Instead, we emphasize that our data do not evaluate treatment responsiveness and therefore do not support sex-specific therapeutic recommendations. The observed differences in IVSD, LV mass index, and stroke volume throughout follow-up primarily reflect sex-related variation in cardiac structure that affects diagnostic interpretation. Whether such structural differences translate into clinically meaningful variation in treatment response cannot be determined from this study and requires prospective evaluation.

Importantly, while renal function was significantly worse in women at baseline and remained lower after 6 months, cardiac biomarkers such as NT-proBNP and troponin showed no significant sex-specific differences over the follow-up period.

To assess whether sex differences in myocardial structure were driven by confounding, we performed multivariable linear regression, particularly showing that adjusting for body surface area and NAC stage attenuated the effect. Clinically, however, such adjusted values are not used. Diagnostic decisions are based on the crude echocardiographic measurements, and these remained clearly and highly different between women and men.

Additionally, there were no significant differences in NYHA functional class between sexes at 6 and 12 months, indicating that while echocardiographic and functional disparities persist, symptomatic burden may be comparable. Our findings suggest that existing diagnostic criteria for ATTR-CM may underdiagnose women, as they often present with lower IVSD, LVMMi, and SV despite advanced disease. These results underscore the need to reassess and potentially modify current diagnostic thresholds to better reflect sex-specific disease expression. In clinical echocardiography, normal values for IVSD and LVMMi are typically indexed and evaluated according to sex, with women exhibiting values approximately 1 mm lower on average.^15^ Yet, in amyloidosis screening, a uniform IVSD cut-off of ≥12 mm is still widely used, regardless of sex. This may disproportionately exclude women with cardiac involvement.

This notion is supported by a recent multicentre study by Aimo et al., who investigated left ventricular wall thickness in women and men with ATTR-CM and demonstrated that wall thickness values indexed to body size, particularly to height²·⁷, more accurately reflect the severity of cardiac involvement than non-indexed measurements.^16^ In that study, the authors proposed replacing the single 12 mm cut-off with sex- or height-adjusted thresholds, corresponding to an approximate diagnostic cut-off of 11 mm for women. Our findings of lower absolute IVSD and LV mass in women despite a comparable symptomatic and biomarker burden are consistent with these results and provide real-world evidence that current non–sex-specific cut-offs may contribute to delayed diagnosis in female patients.

A sex-specific cut-off, e.g. 13 mm for men and 12 mm for women, should be considered, in alignment with established echocardiographic norms. Future studies should also evaluate how many women in amyloidosis cohorts present with IVSD < 12 mm, as these patients may be systematically missed despite having clinically relevant cardiac involvement.

**Figure 4:**
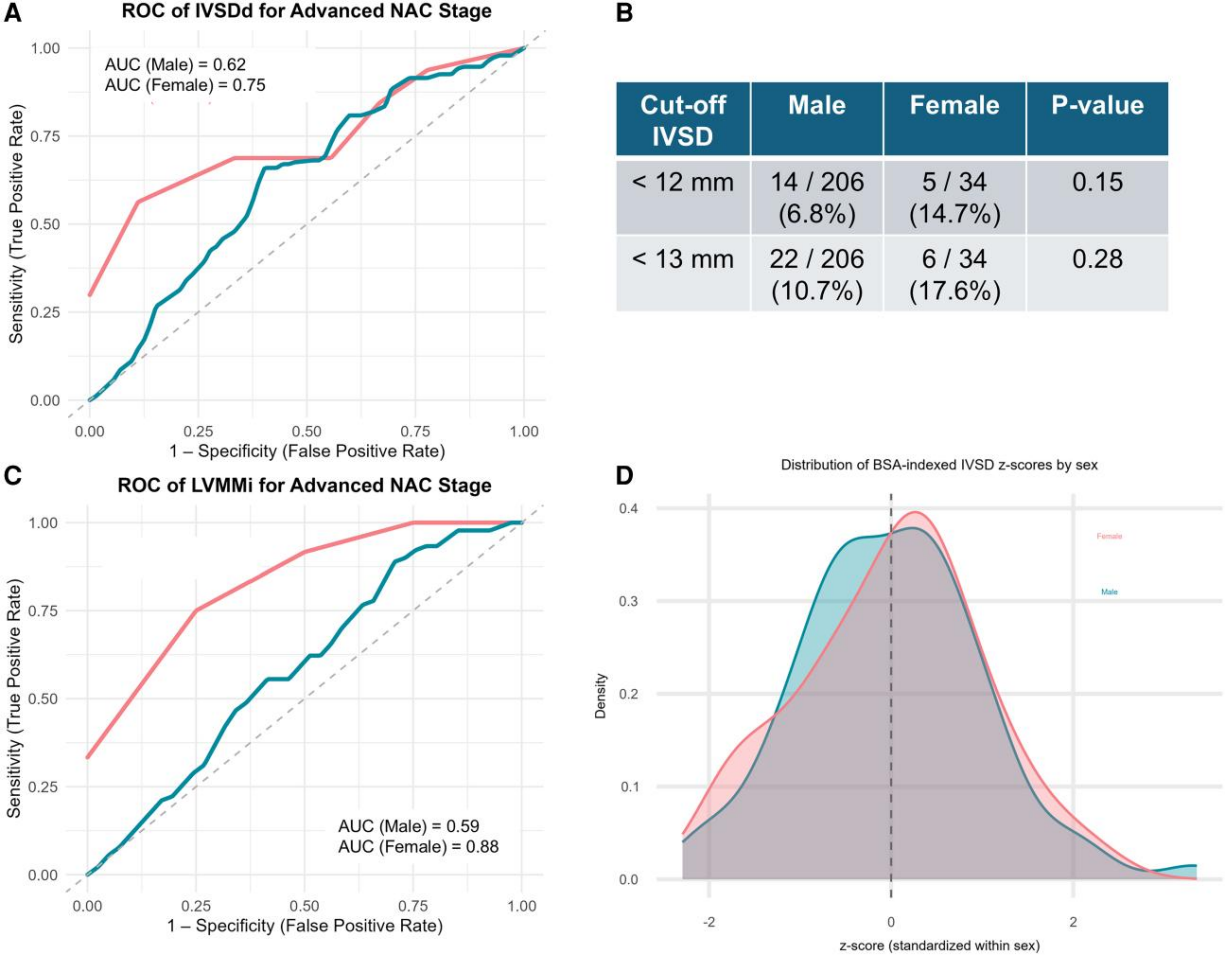
Sex-specific performance of echocardiographic markers for disease staging in ATTR-CM. (*A*) ROC analysis of interventricular septal thickness in diastole (IVSDd) for advanced NAC stage (≥2) showed higher diagnostic discrimination in women (AUC = 0.75) compared to men (AUC = 0.62). (*B*) When applying conventional diagnostic cut-offs (12 and 13 mm), 14.7% of women and only 6.8% of men had IVSD < 12 mm, suggesting that uniform thresholds may under-detect cardiac involvement in women. (*C*) A similar pattern was observed for left ventricular mass index (LVMMi), with a markedly higher AUC in women (0.88) than in men (0.59). (*D*) Distribution of body surface area (BSA)-indexed IVSD z-scores standardized within each sex. Even after BSA adjustment, women showed a leftward shift towards lower standardized IVSD values, confirming smaller relative wall thickness despite comparable disease stage and biomarker burden.

To further evaluate the suitability of current diagnostic cut-offs, we quantified how many patients would fall below the conventional IVSD threshold. Four women (20%) had IVSD < 12 mm, compared with 10 men (3%) (*P* < 0.001), indicating that uniform cut-offs disproportionately miss female patients. Sex-stratified ROC analyses for IVSD and LV mass index (*Figure 4A* and *C*) showed higher diagnostic discrimination in women (AUC = 0.75 and 0.88) than in men (AUC = 0.62 and 0.59). Indexing IVSD to BSA and standardizing within sex (z-scores) revealed a leftward shift in women (*Figure 4B* and *D*), consistent with systematically lower relative wall thickness. These findings support the need for sex- or body-size–adapted diagnostic criteria in ATTR-CM.

Further prospective studies are needed to validate this proposed modification of diagnostic criteria and assess its impact on clinical outcomes. In addition, a deeper understanding of the pathophysiological mechanisms underlying these sex-specific differences is crucial to refining therapeutic approaches and improving prognosis for women with ATTR-CM.

### Limitations

The study is a monocentric retrospective analysis from the Ruhr area, which represents a limitation, as findings also may not be fully generalized to less densely populated regions with limited access to medical facilities. Additionally, there is a risk of reporting bias due to insufficient or incorrect documentation of initial signs and symptoms. The study’s focus on cardiology reflects the typical diagnostic pathway for ATTRwt-CM, predominantly affecting older males with cardiac symptoms, which explains the low representation of ATTRv-CM. Another limitation is the unequal distribution of variant ATTR between women and men (11.8% vs. 2.4%). Although ATTR subtype was included in the multivariable models, phenotype-related differences may still introduce residual bias. Two ATTRv patients (one woman, one man) presented initially with neuropathy and were diagnosed earlier through neurological evaluation or family screening, independent of echocardiographic findings. This may partly explain the earlier detection of some ATTRv cases and creates a diagnostic pathway different from ATTRwt-CM, where diagnosis relies primarily on cardiac assessment. The higher frequency of ATTRv among women may therefore indirectly accentuate the apparent diagnostic gap in female ATTRwt-CM patients. A complete list of TTR variants is provided in Supplementary material online, *Table S5*. Furthermore, the overall number of female patients included was notably low, making comparisons with the substantially larger male cohort difficult and limiting sex-specific conclusions. This raises the question of whether the observed differences truly reflect sex-specific disease manifestations or are at least partly due to underdiagnosis of cardiac amyloidosis in women.

## Conclusion

Sex-specific differences in ATTR-CM persist throughout the disease course, affecting diagnosis and disease progression. Women experience a longer diagnostic delay and exhibit distinct echocardiographic and functional characteristics despite similar symptomatic burden. The findings emphasize the need for sex-specific diagnostic and therapeutic strategies to ensure timely recognition and appropriate management. Future research should prioritize multicentric studies with diverse populations and prospective evaluations of sex-specific diagnostic algorithms to enhance the understanding and management of ATTR-CM in female patients.

## Lead author biography


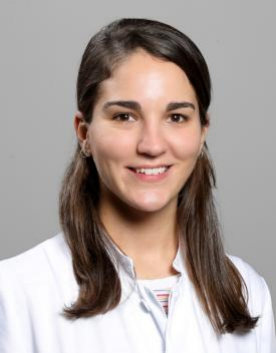


Dr Julia Vogel is a resident physician specializing in cardiology at the Department of Cardiology and Vascular Medicine, University Hospital Essen. Julia’s main interest is heart failure, particularly focusing on cardiac amyloidosis.

## Supplementary Material

oeaf175_Supplementary_Data

## Data Availability

Anonymized data are available from the corresponding author upon reasonable request and in accordance with applicable ethical and data protection regulations.
