# Supplementary material for: Sex-specific differences in cardiac transthyretin amyloidosis: addressing the diagnostic gap in women
Source: Eur Heart J Open. 2025 Dec 26;6(1):oeaf175. doi: 10.1093/ehjopen/oeaf175 (PMC12836091; doi:10.1093/ehjopen/oeaf175)
Supplement: oeaf175_Supplementary_Data [file oeaf175_supplementary_data.zip › Supplementary Table 1 Tafamidis.docx]

|  | **Male** | **Female** | **p-value** |
| --- | --- | --- | --- |
| Tafamidis total, n(%) | 196 (95.1) | 30 (88.2) | 0.14 |
| Tafamidis 20 mg until 2020, n(%) | 15 (7.6) | 2 (5.9) | 0.99 |
| Discontinued, n(%) | 35 (17.9) | 4 (13.3) | 0.79 |
| Frailty, n(%) | 11 (5.6) | 0 (0) | 0.36 |
| Decompensated HF & NYHA IV, n(%) | 20 (10.2) | 4 (13.3) | 0.76 |
| Palliative, n(%) | 2 (1.0) | 0 (0) | 0.99 |
| No follow up, n(%) | 2 (1.0) | 0 (0) | 0.99 |

**Supplementary Table S1:** Tafamidis treatment characteristics stratified by sex. The table summarizes tafamidis treatment rates, use of the 20 mg formulation before February 2020, and reasons for discontinuation among men (n = 206) and women (n = 34). Values are given as absolute numbers (n) and percentages (%).
P-values were calculated using Fisher’s exact test. No statistically significant sex differences were observed for tafamidis treatment rates, dose, or discontinuation reasons (all *p* > 0.05). NYHA, New York Heart Association
