# Supplementary material for: Sex-specific differences in cardiac transthyretin amyloidosis: addressing the diagnostic gap in women
Source: Eur Heart J Open. 2025 Dec 26;6(1):oeaf175. doi: 10.1093/ehjopen/oeaf175 (PMC12836091; doi:10.1093/ehjopen/oeaf175)
Supplement: oeaf175_Supplementary_Data [file oeaf175_supplementary_data.zip › Supplementary Table 2 Multivariable.docx]

| **outcome** | **term** | **estimate** | **conf.low** | **conf.high** | **p.value** | **p_FDR** |
| --- | --- | --- | --- | --- | --- | --- |
| IVSDd | sex female | -1,851 | -4,109 | 0,408 | 0,108 | 0,287 |
| IVSDd | age | 0,062 | -0,060 | 0,184 | 0,320 | 0,426 |
| IVSDd | BSA | 3,774 | -0,558 | 8,105 | 0,087 | 0,287 |
| IVSDd | ATTR_type hATTR | 2,370 | -1,807 | 6,547 | 0,264 | 0,426 |
| IVSDd | NAC | 0,645 | -0,124 | 1,414 | 0,100 | 0,287 |
| IVSDd | CAD yes | 0,017 | -1,408 | 1,443 | 0,981 | 0,981 |
| IVSDd | AF yes | 0,510 | -1,096 | 2,117 | 0,531 | 0,607 |
| LVMMi | sex female | -40,225 | -95,455 | 15,004 | 0,151 | 0,890 |
| LVMMi | age | 0,551 | -1,684 | 2,786 | 0,625 | 0,890 |
| LVMMi | BSA | 14,395 | -76,915 | 105,706 | 0,754 | 0,890 |
| LVMMi | ATTR_type hATTR | 36,191 | -61,102 | 133,485 | 0,461 | 0,890 |
| LVMMi | NAC | 2,522 | -11,274 | 16,317 | 0,717 | 0,890 |
| LVMMi | CAD yes | -10,518 | -36,313 | 15,276 | 0,419 | 0,890 |
| LVMMi | AF yes | -1,537 | -32,726 | 29,653 | 0,922 | 0,922 |

**Supplementary Table S2.** **Multivariable linear regression for sex-specific differences in echocardiographic parameters.**

Models were adjusted for age, BSA, ATTR subtype, NAC stage, CAD, AF, and NYHA functional class. β estimates represent mean differences with 95% confidence intervals (CI); p_FDR denotes *p*-values adjusted for multiple testing (false discovery rate). IVSDd = interventricular septal diameter in diastole; LVMMi = left ventricular mass index; BSA = body surface area; ATTR = transthyretin amyloidosis; NAC = National Amyloidosis Centre stage; CAD = coronary artery disease; AF = atrial fibrillation; CI = confidence interval; FDR = false discovery rate.
