# Supplementary material for: Sex-specific differences in cardiac transthyretin amyloidosis: addressing the diagnostic gap in women
Source: Eur Heart J Open. 2025 Dec 26;6(1):oeaf175. doi: 10.1093/ehjopen/oeaf175 (PMC12836091; doi:10.1093/ehjopen/oeaf175)
Supplement: oeaf175_Supplementary_Data [file oeaf175_supplementary_data.zip › Supplementary Table 3 STROBE Checklist.docx]

| **Item No.** | **STROBE Recommendation** | **Location in Manuscript** |
| --- | --- | --- |
| 1 | Title and Structured Abstract clearly identify study design, setting, and key findings | Title and Structured Abstract |
| 2 | Explain the scientific background and rationale for the investigation | Introduction, paragraphs 1–3 |
| 3 | State specific objectives, including any prespecified hypotheses | Introduction, final paragraph |
| 4 | Present key elements of study design early in the paper | Methods: Study design and population |
| 5 | Describe setting, locations, and relevant dates, including periods of recruitment and data collection | Methods: West German Amyloidosis Center, Essen; 2018–2024 |
| 6 | Give eligibility criteria and sources and methods of selection | Methods: Inclusion of all patients with confirmed ATTR-CM |
| 7 | Clearly define all outcomes, exposures, predictors, confounders, and effect modifiers | Methods: Data collection and variable definitions |
| 8 | For each variable, give sources and details of methods of assessment | Methods: Echocardiography, lab data per ESC guidelines |
| 9 | Describe any efforts to address potential sources of bias | Methods: Statistical analysis; Discussion – Limitations |
| 10 | Explain how study size was arrived at | Methods: Screening of 402 patients, 240 included |
| 11 | Explain how quantitative variables were handled | Methods: Statistical analysis |
| 12 | Describe all statistical methods, including confounding control and sensitivity analyses | Methods: Statistical analysis section |
| 13 | Report numbers of individuals at each stage of study, and reasons for exclusion | Results: Flow diagram (Figure 1) |
| 14 | Give characteristics of study participants and indicate missing data | Results: Table 1 |
| 15 | Report numbers of outcome events or summary measures | Results: Echocardiographic and follow-up outcomes |
| 16 | Present unadjusted and adjusted estimates with precision | Results: Multivariable regression |
| 17 | Report subgroup or sensitivity analyses | Results: Sensitivity analyses (symptom onset vs imaging, ATTRwt subgroup) |
| 18 | Summarize key results with reference to objectives | Discussion: first paragraph |
| 19 | Discuss limitations of the study | Discussion: Limitations |
| 20 | Give a cautious overall interpretation of results | Discussion |
| 21 | Discuss external validity of results | Discussion: Limitations paragraph |
| 22 | Give source of funding and role of funders | Acknowledgements/Funding statement: None |

**Supplementary Table S3. STROBE Checklist for Observational Studies.**

This table summarizes adherence of the present study to the *STROBE (Strengthening the Reporting of Observational Studies in Epidemiology)* recommendations. Each checklist item is mapped to the corresponding section of the manuscript to ensure transparent and comprehensive reporting of study design, methods, results, and interpretation.ATTR-CM, transthyretin amyloid cardiomyopathy; ESC, European Society of Cardiology.
