# Supplementary material for: Sex-specific differences in cardiac transthyretin amyloidosis: addressing the diagnostic gap in women
Source: Eur Heart J Open. 2025 Dec 26;6(1):oeaf175. doi: 10.1093/ehjopen/oeaf175 (PMC12836091; doi:10.1093/ehjopen/oeaf175)
Supplement: oeaf175_Supplementary_Data [file oeaf175_supplementary_data.zip › Supplementary Table 4 Missing Data.docx]

| **Variable** | **Missing total** | **Missing male** | **Missing female** | **Percent total** |
| --- | --- | --- | --- | --- |
| Amyloidosis-type | 0 | 0 | 0 | 0 |
| Age | 0 | 0 | 0 | 0 |
| BSA | 2 | 1 | 1 | 0,83 |
| NYHA (1-4) | 14 | 12 | 2 | 5,8 |
| CHD | 0 | 0 | 0 | 0 |
| Stroke/TIA | 0 | 0 | 0 | 0 |
| Diabetes | 0 | 0 | 0 | 0 |
| Arterial hypertension | 0 | 0 | 0 | 0 |
| AFib | 0 | 0 | 0 | 0 |
| LVEF | 17 | 14 | 3 | 7,08 |
| SV | 69 | 58 | 11 | 28,75 |
| IVSDd | 47 | 38 | 9 | 19,5 |
| LVMMi | 141 | 117 | 24 | 58,75 |
| LA-Vol | 112 | 94 | 18 | 46,67 |
| TAPSE | 23 | 18 | 5 | 9,58 |
| Hb | 2 | 2 | 0 | 0,83 |
| INR | 3 | 3 | 0 | 1,25 |
| eGFR | 2 | 2 | 0 | 0,83 |
| Troponin | 2 | 2 | 0 | 0,83 |
| CRP | 2 | 2 | 0 | 0,83 |
| NT-pro BNP | 8 | 7 | 1 | 3,33 |
| NAC stadium | 2 | 2 | 0 | 0,83 |

**Supplementary Table S4. Missing data by variable and sex**

This table summarizes the number and percentage of missing values for key clinical, echocardiographic, and laboratory parameters, stratified by sex. Missingness was generally low (<5%) for baseline and laboratory variables but higher for echocardiographic measures (e.g., LVMMi, LA-Vol) due to incomplete imaging. The extent of missing data was comparable between men and women.
BSA = body surface area; NYHA = New York Heart Association; CHD = coronary heart disease;
AFib = atrial fibrillation; LVEF = left ventricular ejection fraction; SV = stroke volume;
IVSDd = interventricular septal diameter in diastole; LVMMi = left ventricular mass index;
LA-Vol = left atrial volume; TAPSE = tricuspid annular plane systolic excursion;
Hb = hemoglobin; INR = international normalized ratio; eGFR = estimated glomerular filtration rate;
CRP = C-reactive protein; NT-proBNP = N-terminal pro-B-type natriuretic peptide;
NAC = National Amyloidosis Centre staging
