# Supplementary material for: Sex-specific differences in cardiac transthyretin amyloidosis: addressing the diagnostic gap in women
Source: Eur Heart J Open. 2025 Dec 26;6(1):oeaf175. doi: 10.1093/ehjopen/oeaf175 (PMC12836091; doi:10.1093/ehjopen/oeaf175)
Supplement: oeaf175_Supplementary_Data [file oeaf175_supplementary_data.zip › Supplementary Table 5 Mutations.docx]

**Supplementary Table S5:** Overview of TTR variants.

| Gender | p.Ile88Leu | p.Val142Ile | p.Ser43Asn | p.Leu78His | p.Ala65Thr | p.Arg34Gly |
| --- | --- | --- | --- | --- | --- | --- |
| Male | 1 | 1 | 1 | 0 | 1 | 1 |
| Female | 1 | 1 | 1 | 1 | 0 | 0 |

The Table summarizes all TTR variants identified in the cohort, stratified by sex. Six distinct mutations were observed.
