# Supplementary figures and images for: Sex-specific differences in cardiac transthyretin amyloidosis: addressing the diagnostic gap in women
Source: Eur Heart J Open. 2025 Dec 26;6(1):oeaf175. doi: 10.1093/ehjopen/oeaf175 (PMC12836091; doi:10.1093/ehjopen/oeaf175)

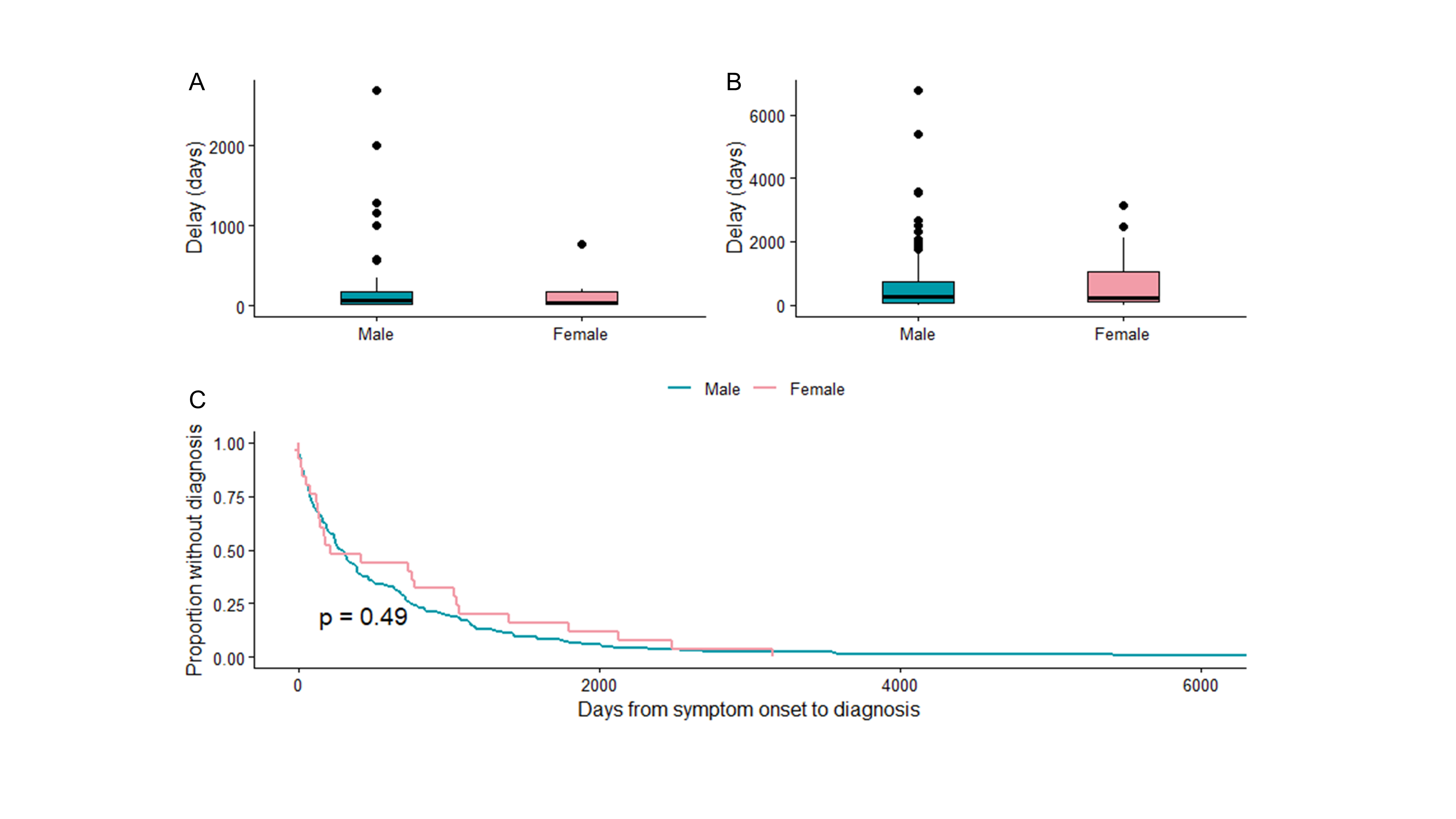

Supplement: oeaf175_Supplementary_Data [file oeaf175_supplementary_data.zip › Supplementary Figure 1 KM Symptom.tif]

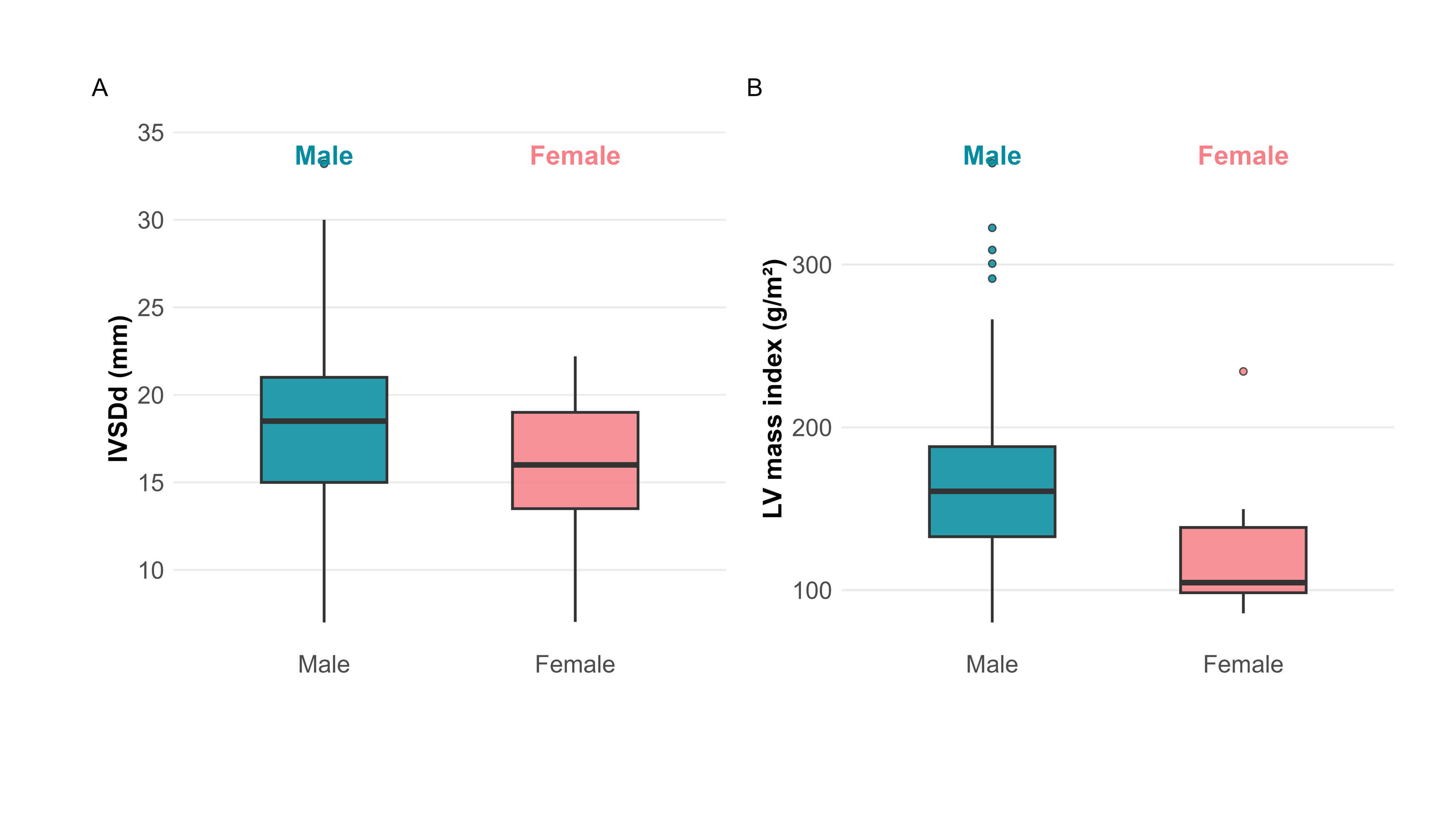

Supplement: oeaf175_Supplementary_Data [file oeaf175_supplementary_data.zip › Supplementary Figure 2 Boxplot IVSD LVMMI.tif]
